# Infraslow oscillations in human sleep spindle activity

**DOI:** 10.1016/j.jneumeth.2018.12.002

**Published:** 2019-03-15

**Authors:** Zsolt I. Lázár, Derk-Jan Dijk, Alpár S. Lázár

**Affiliations:** a Babeş-Bolyai University, Faculty of Physics, RO-400084 Cluj-Napoca, Str. Kogălniceanu Nr. 1, Romania; b Surrey Sleep Research Centre, Faculty of Health and Medical Sciences, University of Surrey, Guildford, UK; c Faculty of Medicine and Health Sciences, University of East Anglia, Norwich, UK

**Keywords:** Sleep, EEG, Sleep spindles, Sigma, Infraslow oscillations

## Abstract

**Background:**

It has previously been reported that EEG sigma (10–15 Hz) activity during sleep exhibits infraslow oscillations (ISO) with a period of 50 s. However, a detailed analysis of the ISO of individually identified sleep spindles is not available.

**New method:**

We investigated basic properties of ISO during baseline sleep of 34 healthy young human participants using new and established methods. The analyses focused on fast sleep spindle and sigma activity (13–15 Hz) in NREM stage 2 and slow wave sleep (SWS). To describe ISO in sigma activity we analyzed power of power of the EEG signal. For the study of ISO in sleep spindle activity we applied a new method in which the EEG signal was reduced to a spindle on/off binary square signal. Its spectral properties were contrasted to that of a square signal wherein the same spindles and also the inter spindle intervals were permutated randomly. This approach was validated using surrogate data with imposed ISO modulation.

**Results:**

We confirm the existence of ISO in sigma activity albeit with a frequency below the previously reported 0.02 Hz. These ISO are most prominent in the high sigma band and over the centro-parieto-occipital regions. A similar modulation is present in spindle activity. ISO in sleep spindles are most prominent in the centro-parieto-occipital regions, left hemisphere and second half of the night independent of the number of spindles.

**Conclusions:**

The comparison of spectral properties of binary event signals and permutated event signals is effective in detecting slow oscillatory phenomena.

## Highlights

• A new method to quantify infra slow oscillations (ISO) in sleep EEG was developed.
• Binary sleep spindling signal (on-off) shows ISO at peak frequency <20 mHz.
• ISO is more prominent in high sigma activity and fast spindles.
• Brain topography, sleep history and sleep stage modulate the features of these ISO.
• The method can detect ISO of various phasic EEG events and cross frequency coupling.

## 1 Introduction

Brain activity is characterized by a multitude of time scales ranging from tens of milliseconds (gamma oscillations) up to hours (e.g. ultradian and circadian modulation) (Buzsáki, 2006). Sleep spindles are phasic oscillatory events corresponding to the sigma band (10–15 Hz) in the human sleep EEG (De Gennaro and Ferrara, 2003, Bódizs et al., 2009). They represent the most salient feature of NREM stage 2 sleep and are closely associated with both physiological and cognitive aspects of sleep (Purcell et al., 2017, Dijk et al., 1993, Bódizs et al., 2005). They introduce two intermediate characteristic time periods, that of the duration of a spindle (∼1 s) and their inter event separation (∼15 s). Beyond these time scales infraslow oscillations (ISO) defined by frequencies in the 0.01–0.1 Hz range have been reported (Achermann and Borbely, 1997, Hughes et al., 2011, Lecci, 2017). The first account of ISO emerged in 1957 (Aladjalova, 1957). The study reported on oscillations with periodicities in the 30–90 s range observed in the electric activity of the neocortex of rabbits. Since then the underlying physiology has been intensely studied including the neural level aspects (Leresche et al., 1991, Steriade et al., 1993, Albrecht et al., 1998, Lüthi and McCormick, 1998, Lüthi and McCormick, 1999), pathophysiology (Steriade and Contreras, 1995, Vanhatalo et al., 2004), and their characteristics in wakefulness and sleep (Bal and McCormick, 1996, Achermann and Borbely, 1997, Mölle et al., 2002, Hughes et al., 2011, Lecci, 2017). Research of ISO relies on a diverse arsenal of data recording techniques including electroencephalography (EEG), electrocorticography and functional magnetic resonance imaging (Nir et al., 2008, Picchioni et al., 2011). ISO in the brain are present at both cortical and subcortical level and in different species. It has recently been shown in both mice and humans that activity in most frequency bands, but primarily in sigma, is modulated at a frequency of 0.02 Hz (Lecci, 2017, Lecci et al., 2017). These ISO were most pronounced in the parietal region and appeared to be connected to memory consolidation and sleep fragility.

The palette of methods for the identification and characterization of ISO scale (0.01–0.1 Hz) periodic and quasi-periodic phenomena in EEG is diverse. Most oscillatory phenomena traditionally targeted by electrophysiology occur at timescales below 5 s. These slower processes are often considered to be temporal variations in the parameters of faster processes. Time-frequency analysis via different integral transforms such as short time Fourier-transforms (e.g., Gábor-transform, S-transform) or wavelet transforms are primary tools for tracing the evolution of the relevant oscillatory activities (Sejdić et al., 2009). The time-frequency representation of the obtained power offers a bird's eye-view on the large scale changes in the properties of fast processes. Subsequent frequency analysis, i.e., power of power calculations, can reveal multiplicative ISO scale periodicities that modulate the amplitude of fast processes. This phenomenon cannot be grasped by methods such as direct current EEG sensitive to low frequency components that combine in an additive manner with the faster components.

Time frequency analysis is employed for identifying and describing phenomena such as cyclic alternating patterns (Parrino et al., 2012) and phasic events such as K-complexes or sleep spindles. The methodological details of the data analysis methods can exert a major influence on the results. The role of reference electrode or the parameters of the algorithm for calculating the power spectral density such as the length of the taper window, the taper function or the applied detrending can all have a major impact on the result.

The discovery and analysis of patterns in the temporal distribution of phasic events in a time series is typically carried out in two independent steps. First, events are located and their individual properties are estimated. Next, the analysis of the inter-event intervals is performed. A wealth of linear, non-linear and statistical methods can be used depending on the specifics of the studied events, e.g., sleep spindle in EEG or QRS complex in ECG, and the type of temporal information to be extracted, e.g., ISO for sleep spindles or heart-rate variability for QRS complexes.

Here we present two methods to identify ISO applied to one dataset. We aimed to study the ISO in both (i) sigma and (ii) automatically detected sleep spindles across NREM stage 2 and slow wave sleep (SWS) and the extent to which they are modulated by brain topography and sleep history. We also proposed to describe the influence of methodological factors such as EEG reference and taper window length used in fast Fourier-transforms (FFT). The rationale behind these analyses is threefold: (i) independent replication of previous results; (ii) test of robustness with respect to change of referencing and taper window length; (iii) comparison of ISO in sigma and sleep spindles. Due to the different mechanisms underlying fast and slow sleep spindles, their contrasting prevalences in the two sleep stages (NREM stage2 and SWS) (Yordanova et al., 2017) and the prominence of high sigma ISO we focused our study on fast spindles. We preferred Fourier analysis over alternative methods because its simplicity, robustness and well established strong and weak points minimize the chance of misinterpretation of the results while favoring reproducibility.

Our power of power approach to ISO in sigma activity is to a large extent similar to that employed in Achermann and Borbely (1997) and Lecci et al. (2017). For the study of periodicities in the incidence or individual properties of sleep spindles we developed a novel method.

## 2 Methods

### 2.1 Laboratory

The study received a favourable opinion from the University of Surrey Ethics Committee and conformed to the Declaration of Helsinki. All participants provided written informed consent before participation in the study. Following an adaptation night, baseline sleep (consecutive night) of 34 young (age: 25.1 ± 3.4, mean ± SD) healthy participants (men: *N* = 16) were analysed. The details of the original protocol are presented in Lázár et al. (2015). An extended monopolar EEG montage was used covering all main brain areas (Frontopolar: Fp1 and Fp2, Frontal: F3 and F4, Temporal: T3 and T4, Central, Parietal: P3 and P4 and Occipital: O1 and O2) was used. The ground and common reference electrodes were placed at FPz and Pz, respectively. Signals from the mastoid derivations [A1 and A2] were also recorded for offline referencing. The PSG data were recorded on Siesta 802 devices (Compumedics, Abbotsford, Victoria, Australia). EEG data were stored at 256 Hz. The low-pass filter was set at 70 Hz and the high-pass filter was set at 0.3 Hz. Electrode impedance was kept below 5 kΩ.

In accordance with previous protocols (Dijk and Czeisler, 1995) sleep staging was performed in 30 s epochs according to the Rechtschaffen and Kales criteria (Kales et al., 1968) by one sleep researcher (ASL) with over 10 years of experience in scoring sleep. In accordance with the standard operating procedures of the Surrey Clinical Research Centre the scoring of ASL was compared to a standard scored data set which showed a concordance exceeding 90%.

All EEG artefacts (e.g. muscle activity/sweating) for each individual EEG channel were visually identified by an experienced scorer and annotated on a three second basis using the EEG browser software Vitascore version 1.5 (Temec Instruments B.V., Kerkrade, The Netherlands). Thereafter all EEG channels were exported for further quantitative EEG analyses.

### 2.2 Data analyses

We approached the analysis of ISO from two directions: (i) Analysis of the spectrogram focusing on the sigma band; (ii) study of the temporal aspects of individually detected spindle events.

#### 2.2.1 Extracting sigma activity

The twelve EEG channels, two sleep stages – NREM2 and slow wave sleep (SWS) – and three different reference setups were analysed independently. All EEG channels were re-referenced using three different systems: (i) alternately to the contralateral mastoid electrodes; or (ii) to their arithmetic mean (linked mastoid); or (iii) to the average of all 12 channels.

Subsequent to re-referencing channels were downsampled to 64 Hz, then split into 4 s segments with 50% overlap providing a time resolution of 2 s. Artefact free segments belonging to the sleep stage of interest (NREM2 or SWS) were retained for spectral analysis. The absolute power spectral density of each 4 s segment was calculated subsequently to applying detrending and Hanning tapering on the entire segment. The frequency resolution of the spectrum was 0.25 Hz.

#### 2.2.2 Infra spectrum of sigma activity

The 2 s × 0.25 Hz resolution time-frequency picture was subject to spectral analysis for identifying longer timescale oscillations. At each frequency contiguous temporal sequences of at least 128 power component values (256 s) were separately subjected to spectral analysis using Welch's periodogram method with 128 point (256 s) windows, 75% (96 points) overlap, mean detrending and Hanning tapering. The final infra power (of power) spectrum was obtained as the length weighted average of the relative (normalized) spectral densities obtained from the different contiguous sequences. Formally:

$$\bar{\mathrm{PSD}}=\frac{\sum_{i}{\mathrm{PSD}}_{i}\times L_{i}}{\sum_{i}L_{i}}\;$$

where *PSD*_*i*_ is the power spectral density (PSD) obtained from the *i*th contiguous segment of length *L*_*i*_.

#### 2.2.3 Spindle detection

Similar to the methodology we described earlier (Atherton et al., 2016) we applied a variant of the spindle detection procedure recommended by O’Reilly and Nielsen (2014). Parameters were adjusted such that it performed optimally when validated against spindle sets identified visually by an expert. The F1-score (harmonic mean of precision and recall) of the validation was ∼0.75 (Warby et al., 2014, Zhao et al., 2017). Briefly, the method followed a threefold thresholding procedure selecting oscillatory transients by their frequency, amplitude and duration. As a final step adjacent events were merged based on a proximity criterion. The algorithm was applied separately on each of the derivations re-referenced to the contralateral mastoid electrodes. The signal was bandpass filtered using a FIR filter of order 603 (for Fs = 256 Hz) parametrized using the Remez exchange algorithm (McClellan and Parks, 1973) (passband: 11.3–15.7 Hz, stopband: 10–17 Hz, stopband attenuation: 1.122 × 10^−5^, passband ripple: 0.0575, density factor: 20). All subsequent steps were carried out on the filtered signal.

The root mean square (RMS) signal was calculated by using a 0.12 s moving window with 1/Fs long time shifts, i.e., with the same time resolution as the original signal. This RMS signal was then smoothed by applying a 1.2 s long Hanning window.

The amplitude threshold was set to the 83rd percentile of the distribution of the so obtained RMS amplitude extracted exclusively from stage 2 sleep.

Subsequent segments with RMS signal exceeding the threshold were fused (i.e., associated with the same spindle) if closer to one another than 1 s and if the duration of the resulting segment remained shorter than 3 s. In order to make the procedure unambiguous the rule was applied recursively starting from the closest pair and fusing a single pair at the time until no more mergeable pairs were found. Segments with duration within the 0.4–3.0 s interval were labeled as spindles.

#### 2.2.4 Spindle event based study

Sleep spindles were classified into slow (11–13 Hz) and fast (13–15 Hz) spindles based on the frequency of the signal's PSD maximum. This rough dichotomization was based on the averaged sigma profile (Fig. 2(a)) presenting a peak in the range of 11–13 over the frontopolar and frontal brain regions and another peak in the range of 13–15 Hz over the central, parietal and occipital brain regions. Similar classification of sleep spindles has also been successfully applied previously (Schabus et al., 2007).

Similarly to the ISO study of the sigma band, contiguous intervals were identified with the following properties: fully contained within a single sleep stage (either stage 2 or SWS), at least 256 s long, start and end with a spindle and contain at least 5 spindles. We shall henceforth refer to these sets as sequence.

**Fig. 1:**
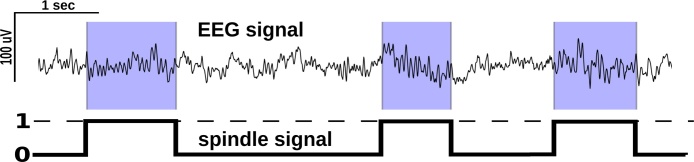
Definition of spindle signal. In segments where spindles were detected by a procedure described in Atherton et al. (2016) and O’Reilly and Nielsen (2014) signal level was set to one while non-spindling segments to zero.

The original signal underlying a spindle sequence was reduced to a binary (square) “spindle signal” of the same length and time resolution. All intra-event (spindling/on state) segments were replaced by one, while inter-event (no spindling/off state) segments were filled in with zeros (see Fig. 1). The spindle signal was downsampled to 4 Hz and its power spectral density calculated using Welch's periodogram method using 256 s Hanning tapered windows with 75% overlap.

**Fig. 2:**
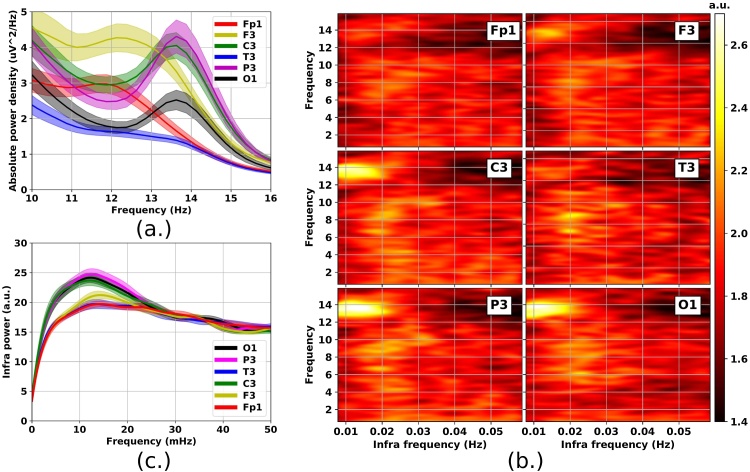
Effect of topography on ISO (relative infra power spectral density of the absolute power spectral density of sleep EEG signals). Average of 34 participants’ baseline NREM stage 2 sleep (contralateral reference). (a) Power spectral density of the sigma band (shaded area = standard error of the mean, SEM). (b) Power spectral density of infra oscillations (arbitrary units). (c) Power spectral density of infra oscillations integrated over the fast spindle sigma band (13–15 Hz) (shaded area = SEM).

In order to correctly identify features attributable to the temporal structure of the alternations between on and off states surrogate data were created. The train of subsequent spindles and the inter-spindle separations were shuffled within a contiguous surrogate EEG segment. The corresponding binary signal was then generated. We shall refer to this surrogate data in all contexts with the label ‘permutated’. The power spectral density of the permutated signal was computed identically to the spindle signal. Due to the strongly decaying dependence of the power on frequency the properties of the spectrum are best revealed on a logarithmic scale. The ratio, i.e., difference on a logarithmic scale, of the power spectral density of the original spindle signal to that of the permutated signal was analysed for all channels and two sleep stages (NREM2, SWS). Within the context of this event based study we shall refer to the PSD(signal)/PSD(permutated) − 1 quantity as excess infra power (EIP).

### 2.3 Simulation of modulated spindle signal

For the correct interpretation of the excess infra power the procedure was repeated using simulated event signals. Using the inversion method (Devroye, 1986) interspindle separations were generated randomly with probability distribution matching that of the real data. The latter was estimated based on the histogram of fast spindles found in channel P3, stage 2 sleep, for all participants. Only events that were considered previously, i.e., those belonging to sufficiently long contiguous intervals contributed to the histogram. Because of the heavy tail of the interspindle distribution, logarithmically spaced binning was used covering the whole range of measured values. A similar procedure was employed for generating the durations of the simulated spindle events except here linear binning was used. The generated *N* separations, *d*_*i*_, *i* = 1, 2, …, *N* were modulated sinusoidally using the formula $d_{i}^{\mathrm{mod}}=d_{i}\left[1+A\sin(2\pi \nu t_{i})\right]$ where $t_{i}=\sum_{j=1}^{i}d_{j}$. The so obtained simulated event signal underwent the same procedure as the experimental data (reshuffling, comparison of spectra for the original and permutated signals in terms of the excess infra power).

The event signal spectrum also contains some method specific features that cannot be associated with real periodicities. By choosing a discontinuous (square shaped) profile for the spindle signal its power spectrum will also include a marked component corresponding to the timescale of spindle duration in the order of s (few tenths of a Hz). This timescale is however far beyond typical ISO frequency values and will not cause any interference with the proposed method. We identified and studied these artefacts by mathematical tools in Appendix A.

## 3 Results

### 3.1 Sigma activity

**Fig. 3:**
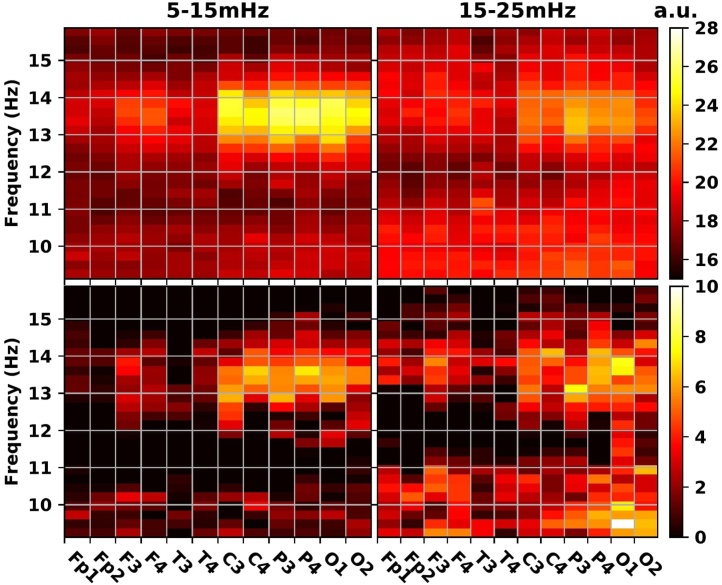
Relative infra power spectral density of the sigma band (9–16 Hz) (see Fig. 2) integrated over the 5–15 mHz (left column) and 15–25 mHz (right column) infra range, respectively, as function of signal frequency and topography. Top heatmaps represent infra powers obtained by averaging across 34 subjects (baseline stage 2 sleep, contralateral referencing). Bottom row contains the corresponding Bonferroni corrected ((16 Hz – 9 Hz)/0.25 Hz × 12 channels = 336 multiplier) *P*-values from one-sample *t*-tests. The mean infra power level (flat spectrum) was used as null hypothesis. Color coding follows the 0.05/*P*-value ratio on base 10 logarithmic scale. Lack of significance appears in black.

In a first step we looked at the relative infra (<0.06 Hz) power spectrum extracted from the time dependence of the EEG absolute power spectrum separately for all frequencies in the 0.5–16 Hz range and across all EEG channels (see Section 2.2.2). In line with an earlier report we show that ISO are most conspicuous in the fast sigma frequency band (13–15 Hz) with a peak around 10 mHz and spreading to 20 mHz (see Fig. 2(b) and (c)) (Lecci et al., 2017). Linked mastoid and average referencing returned results similar to that obtained for contralateral reference (see Section 3.1.1). The 20 mHz periodicity reported previously seems to characterize the oscillations of the subsigma (<11 Hz) band (see Figs. 2(b) and 3 ). Our results also confirm topographic effects on sigma ISO. The observed ISO in fast sigma power was salient over central, posterior and occipital regions (Fig. 2(b) and (c)).

**Fig. 4:**
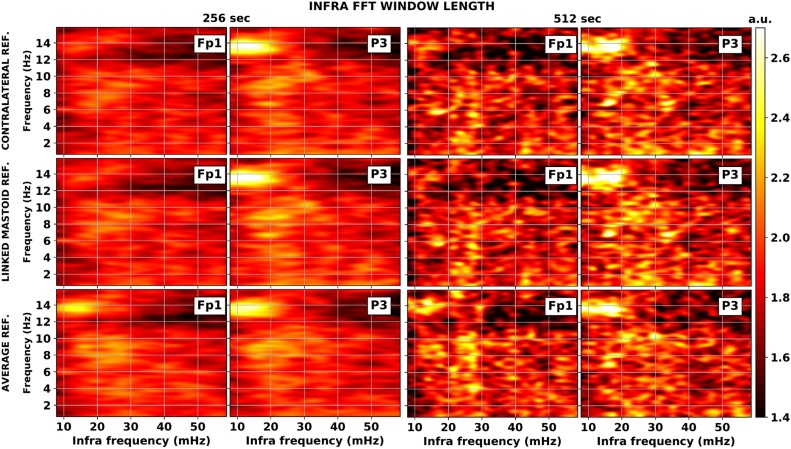
Effect of re-referencing and FFT window size on ISO (relative infra power spectral density of the absolute power spectral density of sleep EEG signals). Average of 34 participants’ baseline NREM stage 2 sleep (contralateral, linked mastoid and average reference). Relative ISO spectrum is calculated from the 2 s temporal resolution of the absolute power spectrum's evolution (0.25 Hz frequency resolution). Minimum length contiguous FFT window for the ISO spectrum is 256 s (to the left) and 512 s (to the right).

In order to clarify the role of the different infra frequencies and gain a better overview of the interaction between ISO and topography the above spectra were integrated over two adjacent infra frequency ranges, namely, the 5–15 mHz and 15–25 mHz, respectively (see Fig. 3). There was an obvious topographic effect along the anterior-posterior axis with the parietal, occipital and central regions dominating the ISO landscape in the 5–15 mHz infra range and the fast sigma band. To assess the statistical significance of the deviation of ISO power from the mean level (flat infra spectrum) we applied a one-sample *t*-test. We found that after Bonferroni correction the ISO were restricted to the fast sigma band and the dominant brain regions (Fig. 3 bottom panels). The ISO power in the 15–25 mHz range (Fig. 3 right panels) was lower but more spread across brain regions compared to the 5–15 mHz range. Notably, ISO in the 15–25 mHz range were statistically significant for the alpha band. In this lower frequency band the prevalence of 20 mHz ISO is also evident from Figs. 2(b) and 4 .

#### 3.1.1 Effect of referencing and larger FFT windows

The topography of EEG features is influenced to a varying extent by the applied reference. Likewise the parametrization of numeric Fourier analysis methods can affect the evaluation of less robust oscillatory phenomena. By increasing the FFT window from 256 s (128 data points) to 512 s (256 data points) the resolution of the infra spectrum doubles. On the other hand, the number of windows to average over will lower yielding a much noisier picture of an already weak effect. This effect is exacerbated by including only sufficiently long contiguous (artefact and sleep stage transition free) segments. Fig. 4 synthesizes the results obtained with the same procedure as in Sections 2.2.1 and 2.2.2 using different FFT window lengths and reference systems. The 512 sec FFT window produces a less clean depiction of ISO which is nevertheless similar to that obtained for the 256 sec FFT window. Fig. 1 in the Supplementary Material offers further insight into this aspect. Another general impression is that there are no apparent specific effects of the referencing on the infra power spectrum. The influence is indirect and mostly attributable to the well documented dependence of the EEG power spectrum on the reference.

### 3.2 Spindle event based study

**Fig. 5:**
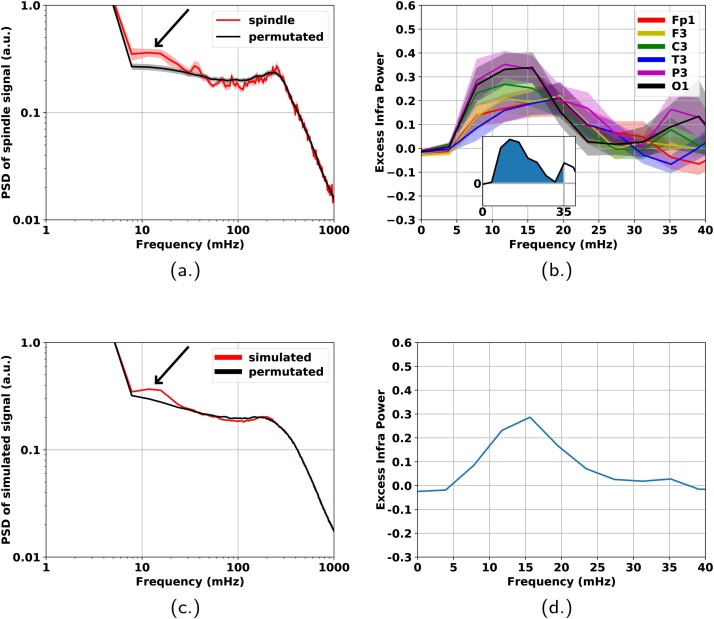
Infra oscillation of fast spindle activity (13–15 Hz) as measured in the power spectral density of the binary spindle signal (see Fig. 1). PSD of the binary spindle signal is compared to that of a surrogate signal obtained from the binary spindle signal by randomly permutating on and off states and the separations in between (see Section 2.2.4). (a) Representative example (P3-A2 channel, NREM2, fast spindles, average across 34 subjects (shaded area = SEM)) for the PSD of the spindle square signal and the corresponding reference signal (NREM2, fast spindles, average across 34 subjects, shaded area = SEM). (b) Excess infra power (EIP), i.e., relative difference between the PSD of spindle and permutated signal. Average of 34 participants. Shaded region illustrates the level of standard error. Inset: The positive (shaded) area between the abscissa and the curve limited to the 0–35 mHz region is also used as a measure of the EIP due to the infra modulation. It is referred to as integrated EIP. (c) PSD of simulated spindle signal (sequences of 100 events with intra event separations modulated at 13 mHz) and the corresponding permutated signal. Continuous lines correspond to the mean of a large statistical ensemble of 1000 similarly prepared simulations therefore standard error is not represented. (d) EIP for the simulated signal.

In view of the prevalence of the fast sigma frequency band in the ISO picture (see Figs. 2 and 3) we have focused on fast sleep spindles in NREM stage 2 sleep (with individual peak frequencies ≥13 Hz). In the proximity of 10 mHz ISO there was a marked excess infra power (EIP). Thus there was a statistically significant difference between the relative PSD of the binary fast sleep spindle signal (see Fig. 1) and the corresponding permutated signal obtained by the shuffling of the on and off states and the separations in between (see Fig. 5(a)). The strong shoulder around 0.2 Hz is a consequence of the square profile of the signal as demonstrated in A. The EIP exhibited a strong deviation from zero with a positive peak around 13 mHz that showed clear topographic effects (see Fig. 5(b)).

**Fig. 6:**
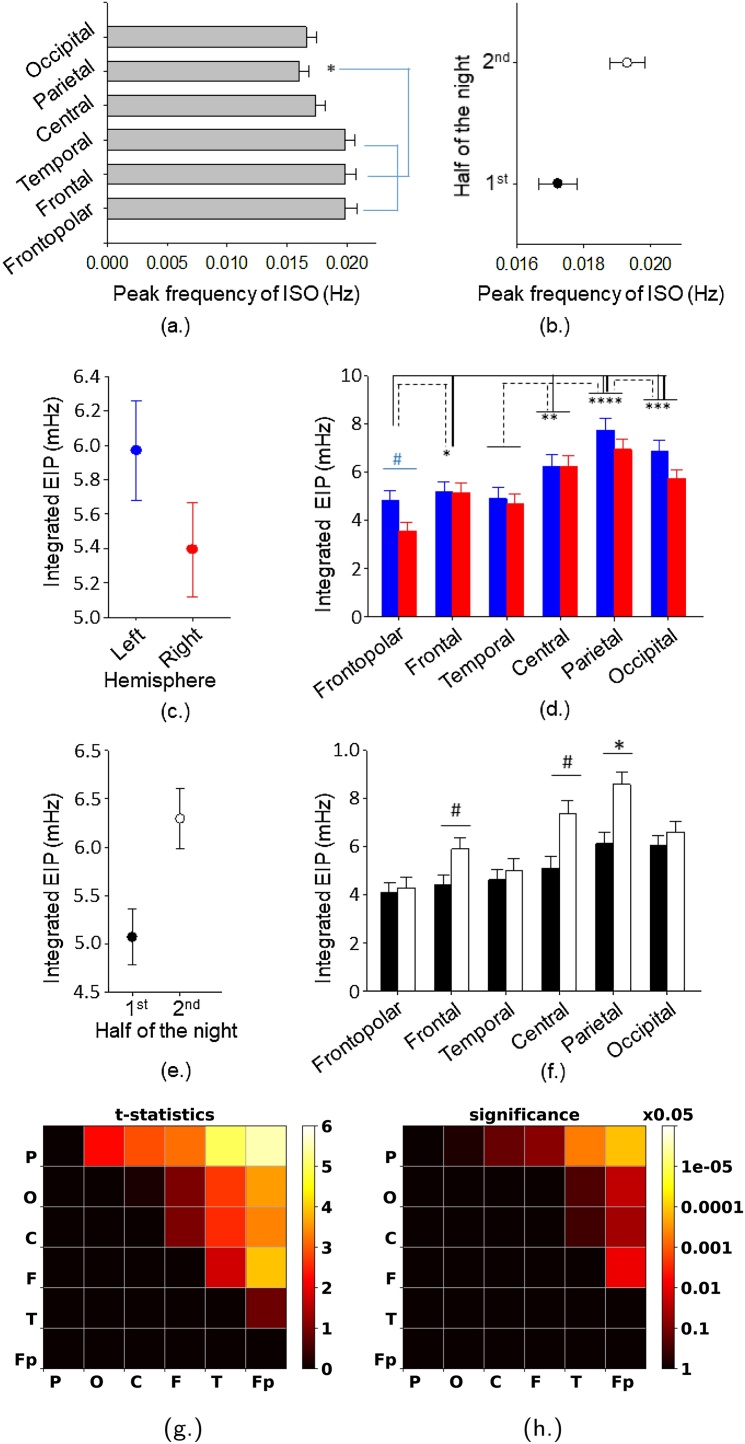
Frequency and power characteristics of infraslow oscillations (ISO) in fast sleep spindle activity (13–15 Hz) in continuous artefact-free NREM stage 2 sleep (34 subjects): effects of brain hemisphere, region and half of the night on peak frequency of infra slow oscillations (ISO) and integrated excess infra power (EIP)(see also Fig. 5). Analyses are controlled for the number of sleep spindles detected in the studied EEG segments and across each EEG derivations (a–f). Least square mean and SEM are indicated. (a) Effect of factor brain region on peak ISO frequency. (b) Effect of factor half of the night on peak ISO frequency. (c) Effect of factor hemisphere on integrated EIP. (d) Interaction between factor brain region and factor hemisphere. (e) Effect of factor half of the night. (f) Interaction between factor half of the night and brain region. Stars indicate significant (adjusted *P* < 0.05) and Tukey-Kramer adjusted post-hoc differences. Stars are indicated only at the second element included in the post-hoc comparison. Dashes indicate trends (adjusted *P* < 0.1). In interaction figures (d and f) significant post-hoc effects are indicated for differences between the brain regions (d and f), the two hemispheres (d) and the two halves of the night (f) but not for the combination of the two factors. Blue bars indicate left hemisphere and red bars right hemisphere (d). Black bars indicate first half of the night and white bars second half of the night (f). (g and h) One sample *t*-test of the integrated EIP (see inset in Fig. 5(b)) difference between all combinations of region pairs. Pairs in reverse order and self comparisons are omitted (in black). *P*-values beyond 0.05 also appear in black. Regions were ordered descendingly based on the integrated EIP.

We investigated the effects of factor hemisphere (left and right), factor brain region (Fp1, Fp2, F3, F4, T3, T4, C3, C4, P3, P4, O1, O2) and factor half of the night (1st and 2nd half) and all interactions using repeated measures ANOVA implemented in a mixed model. First we looked at the EIP peak frequency and we found a significant main effect of brain topography (*F*_5,81.3_ = 4.52, *P* = 0.0011, *f*^2^ = 0.28) and half of the night (*F*_1,65_ = 6.58, *P* = 0.012, *f*^2^ = 0.1) with no significant main effect of factor hemisphere (*F*_1,72.3_ = 0.55, *P* = 0.46, *f*^2^ = 0.008). Tukey-Kramer adjusted post-hoc comparisons revealed that the parietal regions had a significantly (adjusted *P* < 0.05) lower ISO peak frequency compared to the frontopolar, frontal and central brain regions (Fig. 6a). In general the frontal and temporal brain regions showed faster frequency compared to the central and posterior brain regions. The peak frequency of the ISO was significantly faster in the first part of the night compared to the second half (Fig. 6b). None of the interactions were significant (*P* > 0.05).

Second we looked at the integrated EIP (see inset in Fig. 5(b)). We found a significant main effect of factor hemisphere (*F*_1,66.1_ = 6.22, *P* = 0.015, *f*^2^ = 0.094), factor brain region (*F*_5,76_ = 15.90, *p* < 0.0001, *f*^2^ = 1.05) and factor half of the night (*F*_1,70.3_ = 16, *P* = 0.0002, *f*^2^ = 0.23). The left hemisphere presented higher integrated EIP compared to the right hemisphere (Fig. 6(c)) and the second half of the night presented higher integrated EIP compared to the first one (Fig. 6(e)). Based on the significant topographical main effect Tukey-Kramer adjusted post-hoc comparisons indicated that the parietal region had significantly (adjusted *P* < 0.05) higher EIP compared to all other brain regions except the central brain region. Frontopolar region had significantly (adjusted *P* < 0.05) lower integrated EIP compared to all other brain regions except the temporal brain region. Frontal region had significantly (adjusted *P* < 0.05) lower integrated EIP compared to the parietal and occipital brain regions. In general frontal and temporal regions presented lower integrated EIP compared to the central and posterior regions. There was a significant interaction between factor hemisphere and factor brain region (*F*_5,78.6_ = 2.87, *P* = 0.02, *f*^2^ = 0.18) indicating that topographical effects were modulated by hemisphere (Fig. 6(d)). Largest integrated EIP difference was present between the left parietal and the right frontopolar region. The hemispheric difference was largest over the frontopolar region. Similarly there was a significant interaction (*F*_5,76_ = 4.19, *P* = 0.002, *f*^2^ = 0.28) between factor brain region and factor half of the night indicating that topography modulated the sleep dependent changes in integrated EIP. An indeed the half of the night effect was strongest over the parietal, central and frontal brain regions and almost non-existent across the other brain regions (Fig. 6(f)). No other interactions were significant. In a final step we controlled our analyses for the number of sleep spindles, and the number of contiguous artefact free NREM stage 2 spindle sequences as potential confounding factors. With respect to the peak ISO frequency we found that factor brain region lost its significance (*F*_5,88.1_ = 2.02, *P* = 0.083, *f*^2^ = 0.11) whereas factor half of the night remained significant (*F*_1,82.5_ = 5.66, *P* = 0.02, *f*^2^ = 0.07). All EIP reported main effects and interactions remained significant after the statistical control.

The above results were confirmed by an independent and simple comparison of the ISO intensity in the different brain regions yielding a ranking of the regions in terms of the integrated EIP. For all combinations of brain region pairs, *i* and *j* ∈ {P, O, C, F, T, *F*}, the difference in the total from the two hemispheres, *L*_*i*_ + *R*_*i*_ − *L*_*j*_ − *R*_*j*_, (L/R = left/right) was compared to zero through a one sample *t*-test over the 34 data sets. Because of the symmetry properties of the *t*-test reverse order combinations were omitted. According to Fig. 6(g and h) our results show that regions can be ranked as: parietal > occipital > central > frontal > temporal > frontopolar. The weak significance in distinguishing between similarly performing brain regions, e.g., parietal, central and occipital, introduces some ambiguity that is resolved by considering the relationship of these regions with third party regions and also considering the *t*-statistics values.

In order to understand the relationship between this peak's location and the frequency of the modulating infra oscillation a similar procedure was carried out on a simulated square signal modulated at 13 mHz (see Section 2.3). The simulation yields an EIP peak at around 20% beyond the modulating frequency (see Fig. 5(c and d)). This effect is due to the slope of the background as explained in B.When estimating the true ISO frequency from the EIP this correction yields values that are fully consistent with those obtained from the study of the fast sigma frequency band (see Section 3.1 and Fig. 2(c)).

### 3.3 Slow wave sleep

**Fig. 7:**
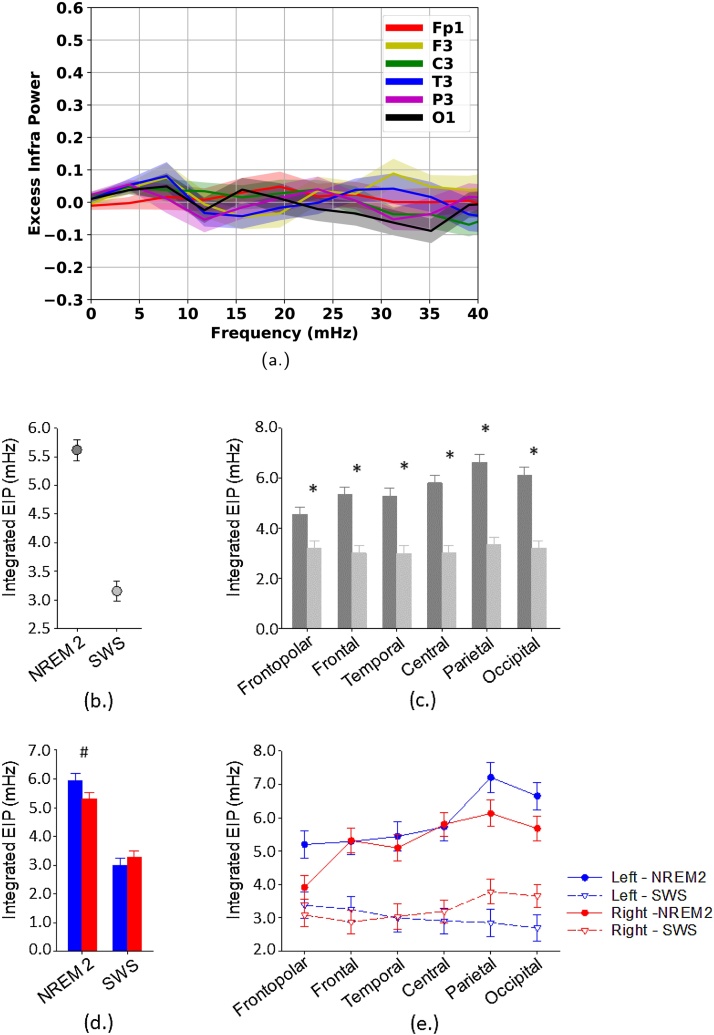
Power characteristics of infraslow oscillations (ISO) in fast sleep spindle activity (13–15 Hz) in continuous artefact-free slow wave sleep (SWS): Comparison with NREM2 (34 subjects). (a) Excess infra power (EIP) of fast sleep spindle activity in SWS. All procedures are similar to those applied for NREM stage 2 sleep (see Fig. 5). (b–e) Comparative analyses of integrated EIP of fast sleep spindle activity across SWS and NREM2, brain hemispheres and regions. Analyses are controlled for the number of sleep spindles detected in the studied EEG segments across each sleep stage and EEG derivation. Least square mean and SEM are indicated. (b) Main effect of factor sleep stage on integrated EIP. (c) Interaction between factors sleep stage and brain region on integrated EIP. Dark gray bars indicate SWS and open gray bars NREM2. (d) Interaction between factors sleep stage and hemisphere on integrated EIP. Blue bars indicate left hemisphere and red bars right hemisphere. (e) Three way interaction between factors sleep stage, brain region and hemisphere on integrated EIP. Stars indicate significant Tukey-Kramer adjusted post-hoc differences (adjusted *P* < 0.05). Dash indicate trends (adjusted *P* < 0.1). For the three way interaction (e) no significant contrasts or trends are indicated.

We next investigated the ISO in sigma activity and fast sleep spindle activity in SWS (Fig. 7). The ISO power in SWS was smaller compared to NREM stage 2 sleep independent of the incidence rate of sleep spindles. For example the EIP measured over the P3 channel was markedly lower in SWS (see Fig. 7(a)) compared to the EIP measured over the Fp1 channel in NREM stage 2 sleep (see Fig. 5(b)) while the two channels presented similar rates of spindle incidence. For a more comprehensive comparison we used similar statistical models as presented above focusing on the integrated EIP only. We included factor sleep stage (NREM2 and SWS) as well as factors brain region and hemisphere in the model. Given that there was no sufficient SWS in the second half of the night factor half of the night was excluded from the model and we analysed the entire night. Provided that the incidence of sleep spindles differs between NREM 2 and SWS we also controlled our analyses for the number of spindles and the number of contiguous EEG segments included in the analyses. We found a significant main effect of factor sleep stage (*F*_1,95.1_ = 108.7, *p* < 0.0001, *f*^2^ = 1.14) and factor brain region (*F*_5,100_ = 2.196, *P* = 0.016, *f*^2^ = 0.15) with significant interactions between sleep stage and brain regions (*F*_5,95.4_ = 2.78, *P* = 0.022, *f*^2^ = 0.16) as well as sleep stage and hemisphere (*F*_1,80.8_ = 5.41, *P* = 0.023, *f*^2^ = 0.07). There was also a three way interaction between all three factors (*F*_5,97_ = 2.63, *P* = 0.028, *f*^2^ = 0.14). The detailed description of the post-hoc contrasts within the interactions exceeds the aim of the current paper. Briefly, the analyses showed SWS has significantly decreased integrated EIP (Fig. 7(b)) compared to stage NREM2 independent of sleep spindle density and the number EEG segments used for analyses. However, this effect was modulated by both brain region (Fig. 7(c)) and hemisphere (Fig. 7(d)) in a way that sleep stage effects were greater over the posterior brain region and the left hemisphere.

## 4 Discussion

We investigated the infra slow oscillations (ISO) in the sleep EEG of healthy humans. We observed that the ISO is most prominent in the sigma frequency range during stage 2 sleep. The frequency of these ISO were in the 0.01–0.02 Hz range, below the previously reported 0.02 Hz (Lecci et al., 2017). A spindle event based analysis revealed that the ISO of fast spindling activity bear the same characteristics as those of sigma power. The intensity of ISO is modulated by brain region and hemisphere with higher values over the centro-parieto-occipital regions and left hemisphere. The ISO are also modulated by sleep history such that ISO power is lower and frequency is higher in the first half of the night. Finally we observed that the ISO are far weaker in SWS than in stage 2.

### 4.1 Comparison with previous studies

Our analyses confirm previous observations but also reveal several new aspects. The present data confirm the existence of ISO in sigma activity (Lecci et al., 2017) although our estimate of its dominant frequency is at ∼0.01 Hz rather than 0.02 Hz. Our observations of sigma activity indicate that the period of this ISO may depend on the sigma frequency range considered. While the ISO frequency of ∼0.02 Hz was present at comparable intensity levels in both the 9–11 Hz and the 12.5–14.5 Hz ranges, at ∼0.01 Hz most of the infra power was due to the ISO of the sigma activity in the 12.5–14.5 Hz range (Fig. 3). In an alternative but equivalent formulation the dominant ISO frequency of the alpha band is around the 0.02 Hz reported by Lecci et al. (2017) while in high sigma this ISO activity is eclipsed by a more intense 0.01 Hz ISO component. The presence of significant ISO in alpha suggests certain overlap in the neurophysiological substrates of these rhythms. This is plausible given the well-known age dependent acceleration of spindle frequency often involving high alpha frequency ranges at younger ages (Purcell et al., 2017). Whereas in previous reports the analysis of ISO was based only on spectral properties of the sleep EEG, we also investigated the presence of ISO in the occurrence of discrete spindle events. We examined here for the first time the modulation of sleep spindles at infra slow frequencies and confirmed the ISO features transpiring from the above study of the sigma activity and that described in Lecci et al. (2017). Peak frequencies of the ISO for the fast sleep spindles (∼0.02 Hz) appear to be somewhat higher than the estimate of the ISO frequency obtained for the sigma band (∼0.01 Hz). Moreover, the ISO frequencies for the fast sleep spindles would only be adjusted upwards by applying the correction due to the background slope of the spindle signal power spectral density (see Fig. 5(a)) as explained in Appendix B. On the other hand this correction is not expected to be significant due to a moderate or no slope around the EIP peak in Fig. 5(a). In summary our data confirms earlier observations of ISO of the sigma activity with slightly longer period and similar topographic dependence of the intensity. The investigation was extended to include the study of ISO of spindling activity and the interplay of factors such as regionality, laterality and sleep time.

### 4.2 Topographical and sleep-time dependent effects

Our results show that brain topography (factor region and hemisphere) and sleep history (factor half of the night) modulate the features of the ISO. The topographic dependence is also consistent with previously reported results (Lecci et al., 2017). When using a contralateral mastoid reference the ISO power in the sigma band exhibits an evident enhancement along the fronto-occipital axis. Similarly, brain topography modulates the ISO of spindling activity with a large effect size for the intensity and a medium effect size for the frequency of the ISO: The ISO over the centro-parieto-occipital region features higher intensities and lower frequencies. The left hemisphere also shows higher ISO intensities whereas it ISO frequency is not markedly different between the two hemispheres. The dominance of the left hemisphere appears to be an effect beyond the general lateralization in spindle activity (Bódizs et al., 2017). We also report medium effect sizes for sleep time on ISO with higher intensity and lower frequency in the second part of the night. Regionality and sleep-time dependent effects are also independent of the number of sleep spindles, the number and length of the continuous artefact free EEG segments used for the estimation of ISO amplitudes.

### 4.3 Sleep stage and spindle density effects

The fact that ISO are prominent in the regions where sigma activity itself is more intensive requires for careful analysis of the dependency of ISO characteristics on intensity of sigma and spindle activity. One approach that can be taken is examining not only the absolute infra spectrum of the absolute sigma activity but also the relative (normalized) counterparts at both levels. The present paper reports on the relative infra spectrum of the absolute sigma activity and ISO were found to be strongly significant even after conservative Bonferroni corrections. The event based method indicates much diminished ISO in sleep spindle activity in SWS compared to NREM stage 2 sleep. We show that this reduction is not due to lower incidence values, suggesting that the mechanisms driving ISO are much diminished in SWS.

### 4.4 Methodological and statistical considerations

We applied robust statistical methods to avoid any spurious positive results. In particular we analyzed the data in two consecutive steps using different approaches. First we applied a more broad-view descriptive approach characterizing the ISO across a large range of EEG frequency bins and multiple brain regions to avoid type 2 statistical error. We then applied Bonferroni correction to tighten the picture and to minimize type 1 statistical error. Based on the findings from the first analysis in our second approach we modeled the studied ISO parameters in a hypothesis driven way evaluating the effects of brain topography and sleep-time on the studied measures implemented in a mixed model. Post-hoc comparisons were controlled for multiplicity. The results from the two analyses yielded similar results provide a convincing picture of the topographical, sleep stage and sleep-time dependence of ISO for sleep spindle activity.

The method of excess power, i.e., the comparison of the sleep spindle signal's spectrum to that obtained by eliminating the temporal structures from the signal, is expected to be applicable for the study of ISO in specific parameters of spindles such as frequency, amplitude or duration. In future analyses the value of the on state in the square signal could be set to a value proportional to the value of the parameter of the corresponding spindle that is suspected to exhibit some large scale temporal structure, e.g., spindle frequency. Any net excess power compared to the same square signal with a constant on state value (e.g. 1), will be attributable to the dynamics of this parameter, e.g., spindle frequency. The presented event based method is not optimized for sleep spindles. It can be directly adapted for the study of ISO in other phasic phenomena like slow waves. With some adjustments the method can be a powerful tool also in the study of cross-frequency coupling (Hyafil et al., 2015, Canolty and Knight, 2010) provided that the interaction of two largely different frequencies, e.g., sigma (∼10 Hz) vs. ISO (∼0.01 Hz), are examined.

On the other hand, the event based method for characterizing ISO is inherently sensitive to the event detection procedure (be it sleep spindles or slow waves) as both low sensitivity and specificity eradicates the temporal patterns in the sequence of events.

### 4.5 Functional considerations and implications

Sleep spindles are intensively studied phenomena in the sleep EEG. Their contribution to sleep protection, sleep dependent memory consolidation and multiple associations with waking cognition and various brain health conditions have been reported repeatedly (De Gennaro and Ferrara, 2003). The presence of ISO in fast sleep spindle activity and its modulation by brain topography and sleep history raises many questions related to their significance for sleep and waking cognition. The fact that ISO of fast sleep spindles are more pronounced in the second half of the night independent of the number of studied sleeps spindles aligns well with it suggested role in the regulation of sleep arousability (Lecci et al., 2017). We point out that from our current analyses based on baseline sleep, we cannot determine whether the differences in ISO between the first and second half of the night are related to sleep dependent or circadian processes. Given that sleep spindles as well as sleep stability exhibit a marked circadian and sleep dependent regulation (Dijk and Czeisler, 1995, Dijk et al., 1997, Wei et al., 1999) it remains to be established to what extent the currently studied ISO is under such circadian and sleep-dependent regulation as well and what the features of that regulation are. The presented event based method opens avenues in exploring the large scale temporal structures of phasic phenomena like sleep spindles or slow waves while targeting specific parameters of these events such as frequency, amplitude or duration.

## 5 Conclusions

We investigated the infraslow oscillations (ISO) in the sleep EEG of healthy humans. We confirmed their existence in the absolute power spectrum of sleep. The ISO landscape is dominated by well-defined peaks of the sigma band positioned at infra frequencies in the 10–20 mHz region. The topographic dependence is also consistent with previously reported results. With contralateral mastoid reference infra oscillations of the sigma band exhibit an evident enhancement along the fronto-occipital axis.

The ISO in fast spindling activity bear the same characteristics as those of the sigma power. Brain topography (factor region and hemisphere) and sleep history (factor half of the night) modulate the features of the studied ISO. Intensity of ISO indicates peak values over the centro-parieto-occipital regions, left hemisphere and second half of the night. The dominance of the left hemisphere appears to be an effect beyond the general lateralization in spindle activity (Bódizs et al., 2017). Regionality and half of the night are also independent of number of sleep spindles and the continuous artefact free EEG segments used for the analyses. The analysis of ISO peak frequency shows similar effects of topography and sleep history. Frontal and temporal regions and the first half of the night feature higher ISO frequencies. These effects are present independently of the number of sleep spindles.

ISO appears not to be as prominent in SWS as in stage 2 sleep. This effect is also modulated by topography and sleep history.

Sleep spindles represent intensively studied phenomena in the sleep EEG given their physiological significance in sleep protection, sleep dependent memory consolidation and multiple associations with waking cognition and various brain health conditions. The presence of ISO in fast sleep spindle activity and its modulation by brain topography and sleep history independent of the number of sleep spindles raises many questions related to their significance for sleep and waking cognition. The fact that ISO of fast sleep spindles are more pronounced in the second half of the night independent of the number of studied sleeps spindles aligns well with it suggested role in the regulation of sleep arousability (Lecci et al., 2017). Given that sleep spindles as well as sleep stability present a marked circadian and sleep dependent regulation it remains to be established to what extent that is present for the currently studied ISO as well and what the features of that regulation are.

The presented event based method opens avenues in exploring the large scale temporal structures of phasic phenomena like sleep spindles, slow waves or ripples while targeting specific parameters of these events such as frequency, amplitude or duration.

## Declaration of interest

None.

## Figures and Tables

**Fig. A8:**
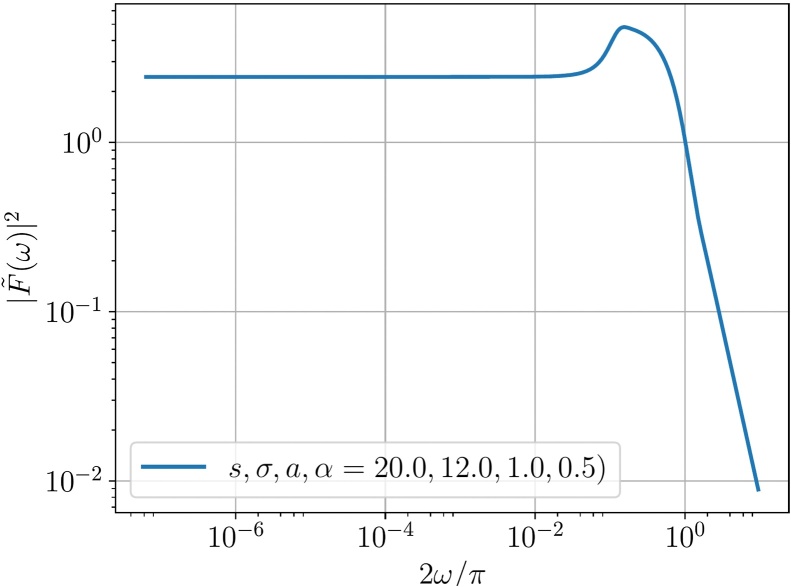
Power spectrum of a theoretical square signal with subsequent square wavelets at varying separations (see Eq. (A.3)). These inter-wavelet distances follow the normal distribution *N*(*s*, *σ*). The widths of the wavelets are also normally distributed (*N*(*a*, *α*)).

**Fig. A9:**
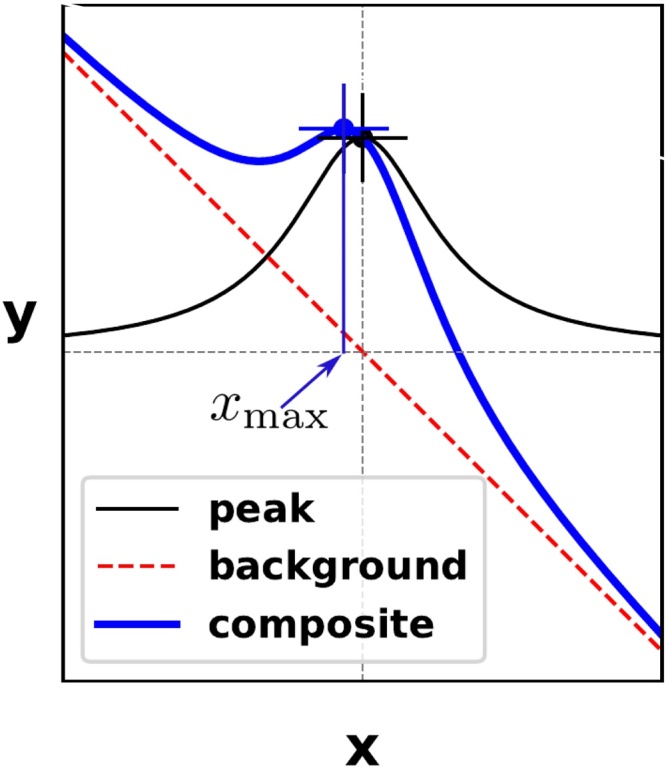
The shift of a Lorentzian peak's (black solid line) maximum location when applied on the top of a slope (red dashed line) (see Eq. (B.1) with *A* = 2.5, *a* = 1). Black crosshair is positioned at the maximum of the Lorentzian peak. Blue crosshair is positioned at the maximum of the composite (Lorentzian + linear) function (blue solid line).

## Appendix A Spectral properties of square signals

We are interested in the basic spectral properties of square signals. In particular, we would like to prove that the shoulder-like peak at 0.2 Hz in the power spectrum of the spindle signal (see Fig. 5(a)) is merely the consequence of choosing square shaped on-states.

Let us construct our signal, *f*, as the superposition of wavelets *φ*:

$$f(t)=\sum_{n\in \mathbb{Z}}\varphi \left(\frac{t-\tau_{n}}{w_{n}}\right)\;$$

where the distances *q*_*n*_ ≡ *τ*_*n*+1_ − *τ*_*n*_ > 0 between subsequent wavelets follow the probability density function (pdf) *d*(*q*). The scale parameter of the individual wavelets is $w_{n}>0$ with pdf h(w). The Fourier-transform of the signal reads

$$F(\omega )=\sum_{n\in \mathbb{Z}}\int_{-\infty }^{+\infty }\varphi \left(\frac{t-\tau_{n}}{w_{n}}\right)e^{i\omega t}dt=\sum_{n\in \mathbb{Z}}w_{n}e^{i\omega \tau_{n}}\Phi (\omega w_{n})\;$$

where Φ represents the Fourier-transform of *φ*. For square shaped wavelets, i.e.,

$$\varphi (t)=\left\{\begin{array}{cc} 1\; & \mathrm{if}\;|t|<1\\ 0\; & \mathrm{if}\;|t|\geq 1 \end{array}\right)\;$$

the corresponding Fourier-transform is

$$\Phi (\omega )=2\frac{\sin\omega }{\omega }.$$

For the Fourier-transform of the signal we get

$$F(\omega )=\frac{2}{\omega }\sum_{n\in \mathbb{Z}}e^{i\omega \tau_{n}}\sin\omega w_{n}.$$

The statistical mean of the power spectral density, i.e., ensemble average over the set of possible locations, *τ*, and scales, w, is

(A.1) $$\begin{array}{cc} \langle {\left|F(\omega )\right|}^{2}\rangle_{} & =\frac{4}{\omega^{2}}\sum_{n,m\in \mathbb{Z}}\langle e^{i\omega (\tau_{n}-\tau_{m})}\sin\omega w_{n}\sin\omega w_{m}\rangle_{\tau ,w}=\\ & =\frac{8}{\omega^{2}}\sum_{n>m\in \mathbb{Z}}\langle \cos[\omega (\tau_{n}-\tau_{m})]\rangle_{\tau }\langle \sin\omega w_{n}\sin\omega w_{m}\rangle_{w}+\frac{4}{\omega^{2}}\sum_{n\in \mathbb{Z}}\langle \sin^{2}\omega w_{n}\rangle_{w}. \end{array}$$

The three constituent expressions can be rewritten as

$$\begin{array}{cc} \langle \cos[\omega (\tau_{n}-\tau_{m})]\rangle_{\tau } & =Re\langle e^{i\omega (\tau_{n}-\tau_{m})}\rangle_{\tau }=Re\langle \prod_{k=m}^{n}e^{i\omega q_{k}}\rangle_{q}=Re\langle e^{i\omega q}\rangle_{q}^{\left(n-m\right)}\;\\ \langle \sin\omega w\rangle_{w} & =\int_{-\infty }^{+\infty }\sin(\omega w)h(w)dw=I\mathrm{m H}(\omega )\;\\ \langle \sin^{2}\omega w\rangle_{w} & =\int_{-\infty }^{+\infty }\sin^{2}(\omega w)h(w)dw=\frac{1}{2}\int_{-\infty }^{+\infty }[1-\cos(2\omega w)]h(w)dw=\\ & =\frac{1}{2}[1-R\mathrm{e H}(2\omega )]\; \end{array}$$

and make use of the connection

$$\langle e^{i\omega q}\rangle_{q}=\int_{-\infty }^{+\infty }e^{i\omega q}d(q)dq=D(\omega )$$

where *H* and *D* are the Fourier-transforms of *h* and *d*, respectively. Substituting the above into Eq. (A.1) we get

(A.2) $$\langle {\left|\tilde{F}(\omega )\right|}^{2}\rangle_{}\equiv \lim_{N\to \infty }\frac{\langle {\left|F(\omega )\right|}^{2}\rangle_{}}{N}=\frac{8}{\omega^{2}}{\left[I\mathrm{m H}(\omega )\right]}^{2}Re\sum_{k>0}D{\left(\omega \right)}^{k}+\frac{2}{\omega^{2}}[1-R\mathrm{e H}(2\omega )]$$

where *N* = 2*M* + 1 is the (odd) number of wavelets. $\tilde{F}$ appears naturally due to the proportionality relationship between the “energy” $\int_{-\infty }^{+\infty }f^{2}(t)dt=\int_{-\infty }^{+\infty }|F(\omega ){|}^{2}d\omega$ of the signal and the number of wavelets. *Uniform distribution of square wavelets* As a test case let us assume that *h*(*x*) = *δ*(*x* − *a*) (identical rectangles with half-width *a*) and *d*(*x*) = *δ*(*x* − 4*a*) (centers placed periodically at 4*a* distance between subsequent wavelets). In these conditions

$$H(\omega )=e^{i\omega a}\;\;I\mathrm{m H}(\omega )=\sin\omega \;\;1-R\mathrm{e H}(2\omega )=2\sin^{2}\omega a\;$$

$$D(\omega )=e^{4i\omega a}\;\;Re\sum_{k>0}D{\left(\omega \right)}^{k}=\frac{1}{2}\sum_{k\in \mathbb{Z}}e^{4i\omega \mathrm{a k}}-\frac{1}{2}\;$$

leading to

$$\begin{array}{cc} \langle {\left|\tilde{F}(\omega )\right|}^{2}\rangle_{} & =4\frac{\sin^{2}\omega a}{\omega^{2}}\sum_{k\in \mathbb{Z}}e^{4i\omega \mathrm{a k}}=\frac{2\pi }{a}\frac{\sin^{2}\omega a}{\omega^{2}}\sum_{k\in \mathbb{Z}}\delta \left(\omega -\frac{k\pi }{2a}\right)=\\ & =\frac{8a}{\pi }\sum_{m>0}\frac{\delta \left[\omega -(2m-1)\frac{\pi }{2a}\right]}{{\left(2m-1\right)}^{2}}. \end{array}$$

The above result is consistent with the expected Fourier-series expansion of a periodic square signal.

*Normal distribution of square wavelets* Let both the inter-wavelet distance and the wavelet scale follow normal distributions, i.e.,

$$d(q)=\frac{1}{\sqrt{2\pi }\sigma }e^{-\frac{{\left(q-s\right)}^{2}}{2\sigma^{2}}}\;\;h(w)=\frac{1}{\sqrt{2\pi }\alpha }e^{-\frac{{\left(w-a\right)}^{2}}{2\alpha^{2}}}\;$$

with Fourier-transforms

$$D(\omega )=e^{i\omega s-\omega^{2}\sigma^{2/2}}\;\;H(\omega )=e^{i\omega a-\omega^{2}\alpha^{2/2}}.$$

Since |D(ω)|<1∀ω∈ℝ∀σ>0

$$Re\sum_{k>0}D{\left(\omega \right)}^{k}=Re\frac{D(\omega )}{1-D(\omega )}=\frac{1}{2}\frac{\cos(\omega s)-e^{-\omega^{2}\sigma^{2/2}}}{\cosh\left(\omega^{2}\sigma^{2/2}\right)-\cos(\omega s)}.$$

Substituting the above into Eq. (A.2) we get

(A.3) $$\langle {\left|\tilde{F}(\omega )\right|}^{2}\rangle \frac{\omega^{2}}{2}=2\sin^{2}(\omega a)e^{-\omega^{2}\alpha^{2}}\frac{\cos(\omega s)-e^{-\omega^{2}\sigma^{2/2}}}{\cosh\left(\omega^{2}\sigma^{2/2}\right)-\cos(\omega s)}-\cos(2\omega a)e^{-2\omega^{2}\alpha^{2}}.$$

If the parameters of the two distributions are chosen such that their relationship resembles those encountered for the sleep spindle signal we get a spectrum shown in Fig. A8. When compared to the spectrum obtained from EEG (see Fig. 5(a)) one can conclude that the pronounced shoulder-like peak is merely the consequence of the square shape of the wavelets.

## Appendix B Maximum shift for a peak on a slope

We are interested in how a peak's position can be determined when overlaid on the top of a linear component (Fig. A9). This bears relevance to finding the dominant frequency in a spectrum in the presence of a linear background. Let us assume our peaked function as being composed additively from a linear and a Lorentzian function both centered on the origin:

(B.1) $$f(x)=-\mathrm{ax}+\frac{A}{1+x^{2}}\;\;a,A>0\;a\ll 1.$$

Close to the peak's maximum, i.e., for |*x*| ≪ 1, the series expansion to second order gives

$$f(x)\approx -\mathrm{ax}+A(1-x^{2}+x^{4}+\cdots )\approx -\mathrm{ax}+A(1-x^{2}).$$

For finding the location of the maximum we look for the zero point of the derivative:

$$f^{\prime }(x_{\max})\approx -a-2{\mathrm{Ax}}_{\max}=0\;\;\to \;x_{\max}\approx -\frac{a}{2A}.$$

We find that the maximum is shifted from zero to *x*_max_ ≈ − *a*/2*A* (see Fig. A9) This shift is more significant for less pronounced peaks on steep slopes. The result is the same if instead of the Lorentzian we use a Gaussian.
