# Supplementary material for: Infraslow oscillations in human sleep spindle activity
Source: J Neurosci Methods. 2019 Mar 15;316:22–34. doi: 10.1016/j.jneumeth.2018.12.002 (PMC6390176; doi:10.1016/j.jneumeth.2018.12.002)
Supplement: Supplementary file 1 [file mmc1.pdf]

SUPPLEMENTARY MATERIAL
Infraslow oscillations in human sleep spindle activity

Zsolt I. Lázár, Derk-Jan Dijk, Alpár S. Lázár

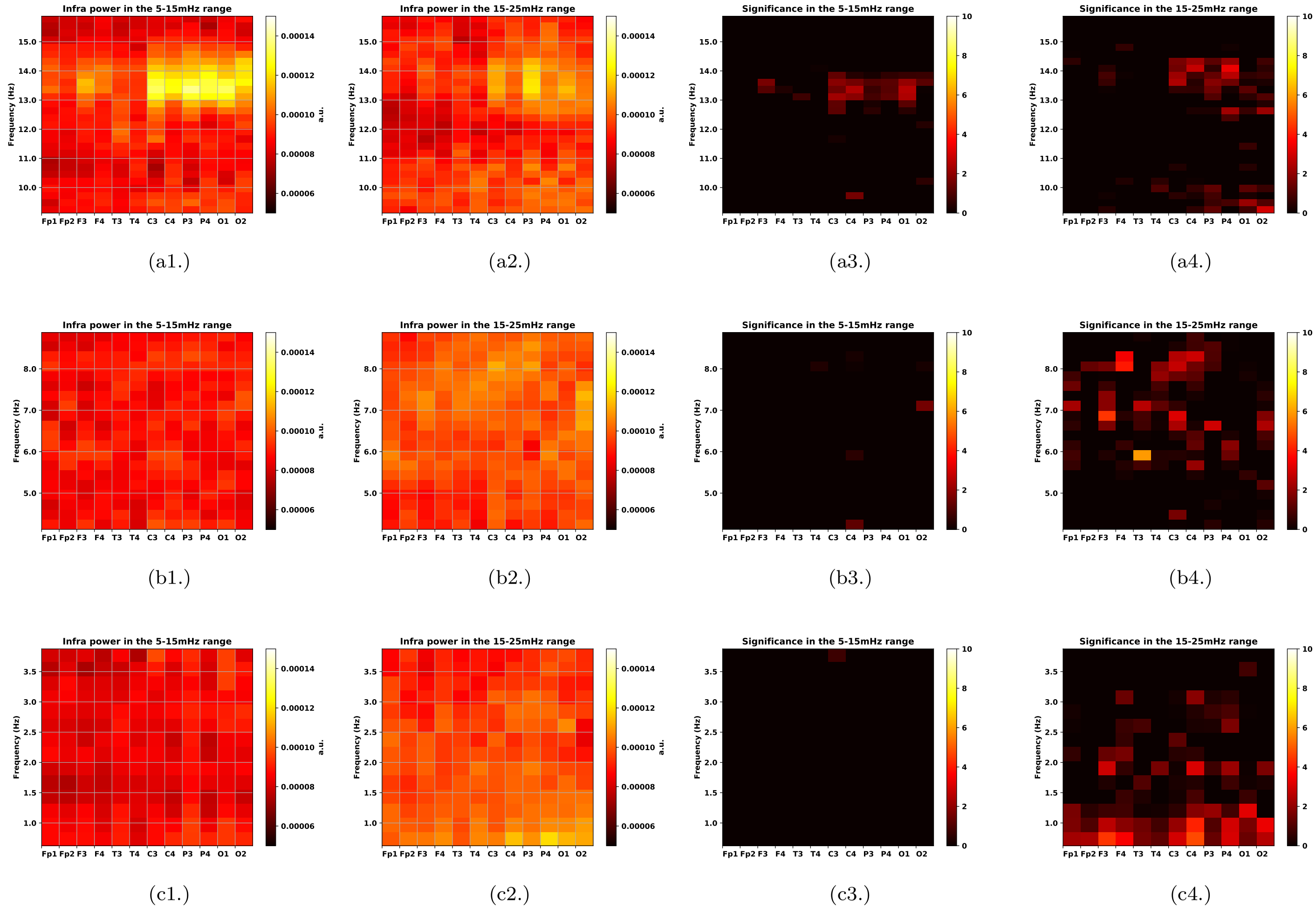

Figure 1: Effect of double size (512 seconds) FFT window on the relative infra power spectrum (see Fig. 4 in the main text) integrated over the 5-15mHz and 15-25mHz infra range, respectively, as function of signal frequency and topography (see also Fig. 3 in the main text). The three rows, a, b and c, correspond to frequency bands, 9-16Hz, 4-9Hz and 0.5-4Hz, respectively. Columns 1 and 2 represent infra powers obtained by averaging over participants' baseline stage two sleep. Columns 3 and 4 contains Bonferroni corrected (e.g., (16Hz-9Hz)/0.125Hz×12 channels = 772 multiplier) P-values from one-sample t-tests. The mean infra power level (flat spectrum) was used as null hypothesis. Color coding follows the P-value/0.05 ratio on logarithmic scale. Lack of significance appears in black. Contralateral referencing was applied. To be compared to the case of 256 seconds FFT window shown in Fig. 3 in the main paper.
